# Effects of Sewage Sludges Contaminated with Chlorinated Aromatic Hydrocarbons on Sludge-Treated Areas (Soils and Sediments)

**DOI:** 10.1100/tsw.2002.880

**Published:** 2002-06-22

**Authors:** Ethel Eljarrat

**Affiliations:** Department of Environmental Chemistry, IIQAB, CSIC, Jordi Girona 18-26, 08034 Barcelona, Spain

**Keywords:** amendment, dioxins, furans, PCBs, sediment, sewage sludge, soil

## Abstract

The fate of PCDDs, PCDFs, and PCBs in sewage sludges after different management techniques — such as agricultural application, land restoration, and marine disposal — was studied. Changes observed in the concentrations, in the ratio between PCDD and PCDF levels, and in the isomeric distribution suggest the influence of the sewage sludge on the sludge-treated areas (soils and sediments). Whereas land application techniques seem to produce no serious environmental consequences, marine disposal practices produce considerable increases in the levels of contamination in marine sediments.

## INTRODUCTION

A number of different directives concerning wastewater sewage sludge have been issued in the last decade. For instance, the promulgation of the Directive COM 91/271 on wastewater treatment requires the installation of treatment systems in all populations exceeding 2,000 inhabitants before the year 2005[1]. On the other hand, the effect of some new directives on treatment and elimination of toxic and dangerous wastes, the EU Directive on agricultural use of sludge (COM 86/278)[2], and the banning of sewage sludge discharges into the sea from 1998[1] all combine to restrict the final fate of sewage sludge. However, there will be much more sludge and further restrictive environmental laws on their use and disposal.

Different sludge management techniques have been used. Disposal of sewage sludge to agricultural and other land is widely practised and is presumed to be environmentally beneficial, but we have found high concentrations of environmentally persistent classes of organic pollutants, such polychlorinated dibenzo-*p*-dioxins (PCDDs), polychlorinated dibenzofurans (PCDFs), and polychlorinated biphenyls (PCBs), in sludges from different countries. The application of sewage sludges to soils is limited by guideline concentrations of heavy metals in the soil through the EU Directive COM 86/278[2]. No similar guidelines exist for organic contaminants in sludges or soils at present. However, in Germany in 1992, the Ordinance on Sewage Sludge[3] established a limit of 100 pg I-TEQ/g of sludge (dry weight, dw) for PCDDs and PCDFs, and 200 ng/g of sludge (dw) for the six PCB congeners (28, 52, 101, 138, 153, and 180) for agricultural purposes. Furthermore, this regulation set an application limit of 5 ton/ha within a period of 3 years.

The aim of this study was to review the effects of the different sewage sludge management techniques on the sludge treated areas (soils and sediments).

## EXPERIMENTAL METHODS

An analytical protocol to determine PCDDs, PCDFs, and PCBs in sewage sludge samples includes the following steps: extraction, cleanup and separation, identification, and quantification. Soxhlet extraction is the most commonly used extraction technique in the world; however, it is time consuming, i.e., extraction times lasting many hours (up to 48), and the solvent consumption is considerable (~300 ml). A number of approaches have been tried to reduce solvent consumption and to speed extraction time. The microwave-assisted extraction (MAE) has been tested by a number of laboratories for the extraction of PCDDs and PCDFs[4]. The considerable saving in extraction time (up to 1 h) and solvent consumption (~30 ml) has made this technique an alternative to the conventional Soxhlet. An accelerated solvent extraction (ASE) has also been developed for extraction of organic micropollutants from solids[5]. ASE uses organic solvents under high temperature and at high pressure resulting in rapid and safe extractions (up to 20 min., ~15 ml).

The cleanup step is based on solid-liquid adsorption chromatography in open columns using a combination of different adsorbents. Among the stationary phases currently available commercially, the most commonly used are modified silica, Florisil, alumina, and different types of carbon (Amoco PX-21, Carbosphere, Carbopack). Recently, automated cleanup systems were developed, based on the use of pressured column chromatographic procedures. A number of studies have evaluated the capability of these automated systems for the PCDD, PCDF, and PCB analyses in biological and environmental samples[6,7].

Ultra-trace determinations of PCDDs/PCDFs are routinely performed using HRGC-HRMS, operating in the electron impact ionisation (electron energy ~38 eV) at 10,000 resolving power[8]. Quantification was carried out by an isotopic dilution technique. Different determinations of PCBs have been developed. A number of countries have chosen to monitor PCBs as a set of seven indicator PCBs (IUPAC Nrs. 28, 52, 101, 118, 138, 153, and 180). These PCBs were analysed by HRGC-ECD (electron capture detector) or by HRGC-LRMS. However, when dioxin-like PCBs (non-*ortho* and mono-*ortho* PCBs) were studied, a HRGC-HRMS with quantification by isotopic dilution method was required[9].

## RESULTS AND DISCUSSION

Different studies of the effects of sewage sludges contaminated with chlorinated aromatic hydrocarbons have been published[10,11,12,13].

## Agricultural Application

The fate of PCDDs, PCDFs, and PCBs in sewage sludges after agricultural application was studied[10]. The study was based on the experiments designed for two soils after applying different rates of sewage sludge. The concentrations of PCDDs, PCDFs, and PCBs in soils were determined before and after the application of sludges, and a general increase was observed with the applied rate, especially in the case of PCDDs. The values of sludge-treated areas were 1.2–11.6 times higher than the respective values of noncontaminated areas. Similar results were obtained by Albrecht et al.[11] who found that the values of sludge-treated areas were 10 times higher than the respective values of noncontaminated areas. Changes observed in the ratio between PCDD and PCDF levels and in the isomeric distribution also suggest the influence of the sewage sludge in the soil. The values of sludge-treated areas were compared with the limits proposed in Germany for agricultural and horticultural land uses[14]. In the case of an initial soil with low contamination, the final soil did not exceed the limit of 5 pg I-TEQ/g; but, in the case of an initial soil with considerable contamination, the final soil exceed this limit which restricts the cultivation of certain vegetables. It should be noted that this study was based on experiments applying high-contaminated sewage sludges (up to 100 pg I-TEQ/g), exceeding the limit application rate of 5 ton/ha over a period of more than 3 years. Despite the incompliance of the German rules, the final soil was suitable for agricultural purposes in the case of initial soil with low contamination levels. Therefore, no serious consequences are expected when these rules are complied with uncontaminated soils.

In general, scientists do not suggest a prohibition of land application but, rather, significantly more restrictive use, i.e., application of clean sludges or application of lesser amounts of less quality sludges. A study of the uptake of dioxins from sewage sludge into various plant species was carried out by Engwall et al.[15]. Their results indicate that the concentrations in carrots were increased when grown in soil amended with high application rates of sludge, but at more realistic applications the increase was absent to small.

## Land Restoration

The increasing amounts of sewage sludge produced and the restrictions in their final fate have increased the search of new possibilities to use this kind of material. On the other hand, the extractive activities, like quarries, which produce big extensions of land without vegetation, constitute an environmental problem that should be solved. The application of sewage sludge–amended soils in order to increase the fertility of these lands can be a solution for this problem and also an interesting application for sewage sludge. However, the evolution of pollutants contained in these sewage sludges in the amended soils should be monitored, mainly considering the high amounts of sludge used compared to those for agricultural purposes. The evolution of PCDDs and PCDFs in sewage sludge–amended soils for land restoration purposes was studied[12]. The results reveal that polluted sewage sludge increases PCDD and PCDF concentration in soils and that these compounds are persistent in the matrix after long periods of time (after 1 year, no evolution is observed and the levels remain constant).

Eduljee[16] described that the ingestion of milk and dairy products obtained from cows grazed on pasture dressed with sludge has the potential to raise the average daily PCDD/F intake via the diet by ~40% if all foods consumed were derived from sludge-amended soil.

## Marine Disposal

Prior to 1998, when sewage sludge discharges into the sea were banned, these discharges were one of the most common methods of sewage sludge disposal. The dioxin contamination in sewage sludge discharges on coastal sediments was evaluated[13]. Sediment samples were analysed at a sewage sludge disposal site as well as in the surrounding areas. The I-TEQ values of sludge-treated areas, 57 pg I-TEQ/g at the dumping site and within a range of 13–48 pg I-TEQ/g near this site, exceeded the safe sediment value of 20 pg I-TEQ/g[17].

The different studies related to the use of sewage sludge showed that marine disposal produced high increases in the sediments at the dumping site as well as in the surrounding areas. The high level of contamination may pose risks to the marine ecosystem. On the other hand, the use of sewage sludge for agricultural purposes seems to be a good practice if there are controls of the contamination levels and rates of the sludges applied. However, further investigation is needed to assess risks to ground and surface water.

## ACKNOWLEDGEMENTS

This paper was presented at the CSIC/ESF workshop *Analysis, Toxicity and Biodegradation of Organic Pollutants in Groundwater from Contaminated Land, Landfills and Sediments*, Barcelona, Spain, 8–10 November, 2001.
